# The impact of COVID-19 pandemic on reported tuberculosis incidence and mortality in China: An interrupted time series analysis

**DOI:** 10.7189/jogh.13.06043

**Published:** 2023-10-13

**Authors:** Yuqi Zhang, Li Zhang, Wenlong Gao, Ming Li, Qiuxia Luo, Yuanyuan Xiang, Kai Bao

**Affiliations:** 1 Institute of Epidemiology and Health Statistics, School of Public Health, Lanzhou University, Lanzhou, Gansu, China; 2 TB Prevention and Control Institute, Lanzhou Municipal Center for Disease Control and Prevention, Lanzhou, Gansu, China

## Abstract

**Background:**

The reported number of cases and deaths from common infectious diseases can change during major public health crises. We explored whether the coronavirus disease 2019 (COVID-19) had an impact on tuberculosis (TB) incidence and mortality in China based on routinely reported TB data.

**Methods:**

We used TB data used from the monthly national notifiable infectious disease reports in China from January 2015 to January 2023. Based on an interrupted time series (ITS) design, we applied Poisson and negative binomial regression models to assess the changes of reported TB incidence and mortality before and during the COVID-19 pandemic.

**Results:**

We found a significant and immediate decrease in the levels of both reported TB incidence (relative risk (RR) = 0.887; 95% confidence interval (CI) = 0.810-0.973) and mortality (RR = 0.448; 95% CI = 0.351-0.572) at the start of COVID-19 outbreak. During the pandemic, the slope of reported incidence decreased significantly (RR = 0.994; 95% CI = 0.989-0.999), while the slope of reported mortality increased sharply (RR = 1.032; 95% CI = 1.022-1.041) owing to an abrupt rise in reported mortality after January 2022.

**Conclusions:**

Both TB incidence and mortality decreased immediately at the start of the COVID-19 pandemic. Over a longer period, the COVID-19 pandemic had contributed to a sustained and more significant decrease in reported incidence, and a delayed but sharp increase in reported mortality.

The emergence of the severe acute respiratory syndrome coronavirus 2 (SARS-CoV-2) led to the rapid spread of the coronavirus disease 2019 (COVID-19) and the ensuing COVID-19 pandemic. In response, many countries took emergency countermeasures by placing restrictions on public gatherings, mandating, medical isolation, dedicating healthcare resources to prevent transmission, and implementing pharmacological interventions [1]. These measures might have effectively reduced the spread of SARS-CoV-2, but they caused an increased strain on healthcare systems and led to unreasonable distribution of healthcare resources, with consequent disruptions in health services, as well as delayed diagnosis, reporting and treatment of other ongoing infectious diseases, including tuberculosis (TB) [2].

The consequences of deferring TB diagnosis and treatment could lead to a transitory, but false reduction in reported incidence (given that patients who were not detected promptly might contribute to a subsequent rise in incidence) and an increase in mortality, which might adversely affect the struggle to end TB [3-7]. For example, TB case notifications in Vietnam decreased by an average of 364 cases per quarter, and the reported mortality in the first quarter of 2020 increased by 0.1% compared to the average for 2016-2019 [4]. Similarly, new TB case notifications in San Francisco in the first four months of the COVID-19 pandemic decreased by about 60% compared with previous years and began to increase from the fifth month, while the reported TB deaths increased by 10% within the first year of the pandemic [5]. Meanwhile, Malawi reported a 35.9% rapid reduction of TB case notifications in the first month after the COVID-19 outbreak, while subsequent TB notifications increased at a proportion of 4.40% per month [6].

Considering the impact of the pandemic, TB-related changes were likely to exist in China as well. Previous studies have reported a marked and short-term decrease in reported TB incidence in the country due to COVID-19 [8,9], yet it is unclear whether this temporary decrease was beneficial for TB control in the long term or how TB mortality was affected. As China is one of the 30 highest-burden TB countries globally, the reduction of TB incidence and mortality could help with ending TB locally, but also positively impact global anti-TB efforts. This emphasises the need to conduct TB surveillance and study the changes in TB incidence and mortality in China before and during the COVID-19 pandemic. We thus aimed to use reported data to determine if and to what extent did the COVID-19 pandemic impacted TB incidence, mortality, and their long-term temporal trends.

## METHODS

### Data source

Following the severe acute respiratory syndrome (SARS) outbreak in 2003, China has strengthened and adopted the China Infectious Disease Reporting Information System, a direct, country-wide network reporting system. As of the date of publication, forty notifiable infectious diseases were reported compulsorily and TB was listed as a Class B infectious disease.

We obtained data on monthly TB case notifications and reported deaths from January 2015 to January 2023 from the monthly national notifiable infectious disease reports announced by the China Bureau of Disease Prevention and Control (Table S1 in the **Online Supplementary Document**). We calculated the monthly reported incidence and mortality of TB (in person-months) as follows:

*Incidence (mortality) = (Number of cases (deaths) per month/Average population for the year)* × *100 000/100 000*

### Study design

We implemented an interrupted time series (ITS) design to assess the COVID-19 pandemic’s impact on reported TB incidence and mortality in China. The ITS design is an evaluation approach with strong internal validity, widely used to assess the effect of population interventions or exposures, especially those related to health outcomes at the population level [10]. It estimates whether an intervention or exposure is effective by comparing the level or slope changes of outcome variables before and after its implementation[11,12]. The level change represents the transitory impact of intervention or exposure on outcomes, while slope change indicates its enduring impact on the long-term trend of outcomes.

### Ethics approval and consent to participate

The Ethics Committee, School of Public Health, Lanzhou University (IRB20103001) approved the study. The data obtained from the China Notifiable Infectious Disease Reports did not involve any individual/identifiable information and did not require informed patient consent.

### Study period, intervention, and outcomes

We defined the intervention in this study’s ITS design as the COVID-19 pandemic in China. Thus, we considered two periods in the study: the pre-COVID-19 period from January 2015 to December 2019 and the COVID-19 pandemic period from January 2020 to January 2023. Our outcomes of interest were the monthly reported TB incidence and mortality.

### Statistical methods

#### Linear regression model for calculating expected incidence and mortality of tuberculosis

We first fitted a linear regression model using historical TB data from 2015 to 2019 to predict the expected incidence and mortality of TB without the impact of the COVID-19 pandemic between January 2020 to January 2023 and compare them with the reported rates, and then generated the counterfactual plots to demonstrate the difference between expected and reported rates. The adopted linear regression model could capture both seasonal variation and year-to-year trends in incidence and mortality, as detailed by Karlinsky and Kobak [13].

#### Interrupted time series analysis

We proposed the level and slope changes model to analyse the transitory and long-term impacts of the COVID-19 pandemic on reported TB incidence and mortality in China simultaneously, as detailed in prior studies [11,12]. Given that the outcomes were the count data by month, we employed a Poisson regression model to conduct the ITS analysis and used the log-transformed population as an offset variable to convert the outcomes into rates and mitigate the impact of population changes over time [14].

#### Methodological issues in the ITS analysis

Overdispersion can result in a decrease in the standard error of estimates and an increase in the false positive rate of a parametric test [15]. Therefore, we set the scale parameter to ×2 in Poisson regression models to adjust to it accordingly. Furthermore, most infectious diseases have their own seasonal pattern. We adjusted to seasonality by setting Fourier terms consisting of 2 sine/cosine pairs in the models. Finally, autocorrelation frequently affects the outcome accuracy in time series data, but can be addressed by controlling seasonality [16]. If autocorrelation still existed after adjusted to seasonality, a heteroskedasticity and autocorrelation consistent (HAC) variance estimation method could be used to weaken its effect.

### Statistical analysis

We adopted negative binomial regression models adjusted to the autocorrelation and seasonality to conduct sensitivity analyses and assess the robustness of the results. We used relative risk (RR) and its 95% confidence interval (CI) in the Poisson and negative binomial regression models as the measures of effect size, with statistical significance set at *P* < 0.05. All models in the ITS analysis were compared by means of Akaike information criterion (AIC) values.

We constructed the linear regression model in Python, version 3.10 (Python Software Foundation, Wilmington Delaware, USA) and performed other analyses in Stata 14.0 (StataCorp, College Station, TX, USA) (Codes 1 and 2 in the **Online Supplementary Document**).

## RESULTS

### Characteristics of reported tuberculosis incidence and mortality before and after the COVID-19 outbreak

There were 5 545 301 TB case notifications in China prior to the COVID-19 outbreak in January 2020 (ie, between January 2015 and December 2019), with an average monthly incidence of 6.6276 per 100 000 person-months and 10 231 reported deaths with an average monthly mortality of 0.0122 per 100 000 person-months (**Table 1**). We observed a decline in overall reported incidence exhibited with distinct seasonality, characterised by a peak between March and August and a trough from September to February (Figure S1 in the **Online Supplementary Document**). The reported mortality showed a slow upward trend and a relatively slight seasonal fluctuation with two small decreases occurring in May to August and November to February per year **(**Figure S2 in the **Online Supplementary Document**).

**Table 1:** Tuberculosis case notifications (incidence) and reported deaths (mortality), before and during COVID-19 pandemic*

	January 2015 to December 2019 (before COVID-19)	January 2020 to January 2023 (with COVID-19)	January 2020 to January 2023 (without COVID-19)‡
Total TB case notifications†	5 545 301	2 470 966	3 105 133
Total TB deaths†	10 231	7267	8081
Average monthly TB case notifications	92 422	66 783	83 923
Average monthly TB deaths	171	196	218
Average monthly TB incidence	6.6276	4.7302	5.9441
Average monthly TB mortality	0.0122	0.0139	0.0155

COVID-19 – coronavirus disease 2019, TB – tuberculosis

*Case notifications presented as numbers; incidence and mortality presented as values per 100 000 person-months.

†Total TB case notifications and deaths calculated by adding the reported monthly numbers, not directly reported data.

‡Values presented as the predicted values if there was no COVID-19 pandemic.

However, when COVID-19 first emerged in January 2020, there was a sudden drop in reported TB incidence from 5.0884 to 4.7964 per 100 000 person-months and TB mortality from 0.0163 to 0.0101 per 100 000 person-months (Table S1 in the **Online Supplementary Document**). We did not observe such sharp decreases during previous seasonal fluctuations. Subsequently, in the COVID-19 pandemic period, reported incidence continued to decrease with the average monthly rate dropping from 6.6276 per 100 000 person-months before the COVID-19 pandemic to 4.7302, while reported mortality did not decrease consistently, and its average monthly rate had increased from 0.0122 to 0.0139 per 100 000 person-months (**Table 1**). Notably, reported deaths from TB in 2020 and 2021 had been lower than that before the pandemic, but suddenly increased by 81% (168 to 304) in January 2022 and remained at a high level until January 2023.

### Expected TB incidence and mortality without COVID-19 pandemic

**Figure 1:**
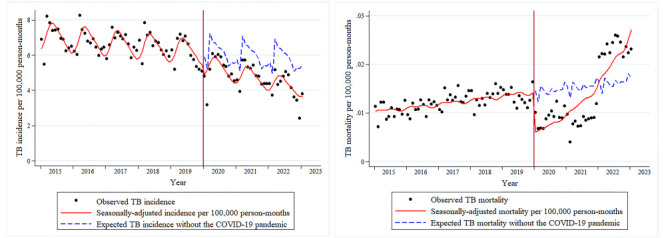
Counterfactual plots of monthly reported TB incidence and mortality in China from 1 January 2015 to 31 January 2023. The blue dashed lines represent the predicted TB incidence and mortality without the impact of the COVID-19 pandemic, using the liner regression models. The red lines were the reported TB incidence and mortality modelled by the adjusted Poisson regression models. TB – tuberculosis, COVID-19 – coronavirus disease 2019.

We further attempted to predict the expected TB case notifications and deaths in China between January 2020 and January 2023 had the pandemic not occurred; we found that there would have been 3 105 133 case notifications and 8081 deaths, with an average monthly incidence of 5.9441 and an average monthly mortality of 0.0155, respectively (**Table 1**). During the 37 months of the COVID-19 pandemic, the monthly expected incidence consistently surpassed the reported incidence, while the expected mortality showed a reversal in January 2022, becoming lower than the reported mortality (Table S2 in the **Online Supplementary Document**). We generated counterfactual plots (**Figure 1**) which showed the differences between reported incidence and mortality and expected incidence and mortality respectively.

### Impact of COVID-19 pandemic on reported TB incidence

**Figure 2:**
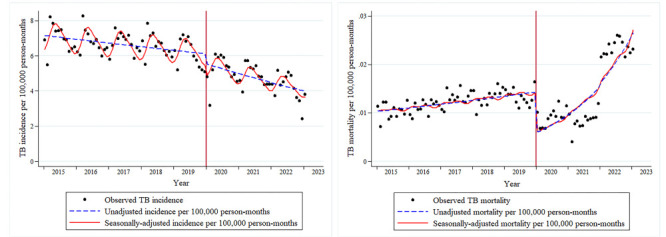
Temporal trends in monthly reported TB incidence and mortality in China from 1 January 2015 to 31 January 2023. These two plots were generated by using the results of the adjusted and unadjusted Poisson regression models. TB – tuberculosis.

As there was overdispersion (mean = 92 421.68, variance = 97 381 094.93) and seasonality in data on TB case notifications (Table S3 in the **Online Supplementary Document**), we applied an adjusted Poisson regression model, the results of which showed a level RR of 0.881 (95% CI = 0.817-0.951, *P* = 0.001) and a slope RR of 0.995 (95% CI = 0.991-0.998, *P* = 0.001) (**Table 2**). Moreover, the goodness-of-fit of the Poisson model adjusted to overdispersion and seasonality (AIC = 644.87) outperformed the unadjusted model (AIC = 1147.03). Overall, the COVID-19 pandemic caused a sudden decrease in the level of reported TB incidence in January 2020 and exacerbated the previous downward trend in reported incidence from then until January 2023 (**Figure 2**).

**Table 2:** Modelled effects of COVID-19 pandemic on reported tuberculosis incidence in China from 1 January 2015 to 31 January 2023

Models	Variables	RR (95%CI)	*P*-value
Unadjusted Poisson regression model (AIC = 1147.03)	Incidence level	0.904 (0.902-0.907)	<0.001
	Incidence slope	0.994 (0.993-0.994)	<0.001
Poisson, adjusted to overdispersion (AIC = 1147.03)	Incidence level	0.904 (0.820-0.997)	0.043
	Incidence slope	0.994 (0.990-0.998)	0.003
Poisson, adjusted to overdispersion and seasonality (AIC = 644.87)	Incidence level	0.881 (0.817-0.951)	0.001
	Incidence slope	0.995 (0.991-0.998)	0.001
Negative binomial model, adjusted to seasonality (AIC = 24.77)	Incidence level	0.887 (0.821-0.959)	0.003
	Incidence slope	0.994 (0.991-0.997)	<0.001
Negative binomial model, adjusted to autocorrelation and seasonality (AIC = 24.77)	Incidence level	0.887 (0.810-0.973)	0.011
	Incidence slope	0.994 (0.989-0.999)	0.024

COVID-19 – coronavirus disease-2019, RR – relative risk, CI – confidence interval, AIC – Akaike information criterion

### Impact of COVID-19 pandemic on reported TB mortality

We also found overdispersion (mean = 170.52, variance = 802.36) and seasonality in the reported TB mortality data (Table S3 in the **Online Supplementary Document**). After adjusting to both in the Poisson regression model, we observed a level RR of 0.416 (95% CI = 0.346-0.501, *P* < 0.001) and a slope RR of 1.035 (95% CI = 1.029-1.042, *P* < 0.001) (**Table 3**). Similarly, the goodness-of-fit of the adjusted model (AIC = 14.15) was better than that of the unadjusted one (AIC = 14.17). The emergence of COVID-19 caused a marked decrease in the level of reported TB mortality, but the slope of reported mortality increased during the COVID-19 pandemic (**Figure 2****)**.

**Table 3:** Modelled effects of COVID-19 pandemic on reported tuberculosis mortality in China from 1 January 2015 to 31 January 2023

Models	Variables	RR (95%CI)	*P*-value
Unadjusted Poisson regression model (AIC = 14.17)	Mortality level	0.422 (0.395-0.452)	<0.001
	Mortality slope	1.035 (1.033-1.038)	<0.001
Poisson, adjusted to overdispersion (AIC = 14.17)	Mortality level	0.422 (0.353-0.506)	<0.001
	Mortality slope	1.035 (1.028-1.042)	<0.001
Poisson, adjusted to overdispersion and seasonality (AIC = 14.15)	Mortality level	0.416 (0.346-0.501)	<0.001
	Mortality slope	1.035 (1.029-1.042)	<0.001
Negative binomial model, adjusted to seasonality (AIC = 12.48)	Mortality level	0.448 (0.379-0.530)	<0.001
	Mortality slope	1.032 (1.025-1.038)	<0.001
Negative binomial model, adjusted to autocorrelation and seasonality (AIC = 12.48)	Mortality level	0.448 (0.351-0.572)	<0.001
	Mortality slope	1.032 (1.022-1.041)	<0.001

COVID-19 – coronavirus disease-2019,– relative risk, CI – confidence interval, AIC – Akaike information criterion

### Sensitivity analyses

Despite adjusting to seasonality of both reported TB incidence and mortality in the Poisson regression models, autocorrelation still persisted and was only improved slightly (Figures S3-S6 in the **Online Supplementary Document**). To address this, we applied negative binomial regression models adjusted to autocorrelation; their level RRs (TB incidence: 0.887, TB mortality: 0.448) and slope RRs (TB incidence: 0.994, TB mortality: 1.032) were similar to those obtained from the Poisson regression models (**Table 2** and **Table 3**), further supporting the robustness and reliability of our findings. Compared with Poisson regression models, the negative binomial regression models had the lowest AIC value (incidence: AIC = 24.77, mortality: AIC = 12.48).

## DISCUSSION

Using an ITS design based on the reported TB data obtained from national notifiable infectious disease reports, we evaluated the impact of the COVID-19 pandemic on TB incidence and mortality, and their long-term trends in China during the COVID-19 pandemic (January 2020 to January 2023). Previous studies [8,9] had suggested that the implementation of stringent COVID-19 prevention measures in 2020 had effectively reduced the reported TB incidence in China, with one [9] predicting a sustained downward trend in reported incidence after 2020 if these measures persisted, but at a decelerating pace. However, we found that reported TB incidence significantly and immediately decreased after the COVID-19 outbreak, while showing a more rapid downward trend than before 2020, in contrast to the findings of the previous study. We also found a notable reduction in reported TB mortality in the early stages of the pandemic, but an upward trend over a longer period. The counterfactual plots (**Figure 1**) derived from expected incidence and mortality from January 2020 to January 2023 also highlighted the same changes in TB caused by the COVID-19 pandemic.

Reported TB incidence in China rapidly decreased from 5.0884 to 4.7964 per 100 000 persons in the first month of the pandemic (January 2020), without a significant recovery in reported incidence afterwards. Simultaneously, the long-term trend had decreased more sharply compared with the previous five years. The pandemic led to the concerning trends of stringent lockdown, missed or at least delayed diagnosis among newly-infected TB patients, and reduced proportions of the population seeking timely medical care, which might have caused the avoidance of ongoing disease reporting and consequent decrease in reported TB incidence [6,17]. However, with the gradual recovery of the healthcare system and growing awareness of COVID-19, it seemed unlikely these reasons could explain the long-term decline in reported incidence. We hypothesised that there were other causes for the long-term decline. First, the preventive measures for COVID-19 decreased the risk of TB transmission. During the COVID-19 pandemic, all provinces in China successively implemented non-pharmaceutical preventive measures such as lockdown or masking and isolation mandates for COVID-19, which were also effective in limiting the transmission of TB and other respiratory infectious diseases. Given the relatively long interval between TB infection and symptom onset, it was impossible to demonstrate an immediate reduction in reported incidence in the first month of preventive measures implementation, but it was more likely to affect its long-term trend. Second, during the COVID-19 pandemic, local governments around the country had organised large-scale disinfection, especially in closed and high-traffic public places. Common disinfectants, such as chlorine-containing disinfectants, 75% ethanol, hydrogen peroxide, and hypochlorous acid had good coronavirus inactivation effects, which were equally effective for *Mycobacterium tuberculosis*. With the dissemination of this knowledge on disinfection, people were more attentive of hand hygiene and disinfection of their living environments, which helped reduce the risk of exposure to *Mycobacterium tuberculosis* and in turn led to decreased reported TB incidence over the long-term period. The beneficial prevention against COVID-19 had promoted a sustained decrease in reported TB incidence. However, as of January 2023, medical services in China were still affected by the COVID-19 pandemic, and it remained unclear whether the decrease in reported incidence was due to beneficial prevention or low reporting. To determine this, further studies are needed for the period after the COVID-19 pandemic in China.

Previous studies from other countries found that the reported TB mortality increased following the COVID-19 outbreak [5,18]. The Global Tuberculosis Report 2021 stated that there were approximately 1.5 million TB deaths worldwide in 2020 – an increase of more than 100 000 compared to 2019 [7]. Unlike them, we found that reported mortality in China decreased significantly rather than increased when COVID-19 emerged (RR = 0.448; 95% CI = 0.351-0.572) (**Table 3**). Many studies have found that the weakened immune system of TB patients could increase susceptibility to COVID-19, and the potential interaction between the two diseases could result in a higher risk of mortality for TB patients infected with COVID-19 [1,19-21]. Furthermore, the mortality associated with COVID-19 was significant during the early stages of the COVID-19 pandemic. Some severely TB-infected patients died from COVID-19, which could have caused the misclassification of individuals as dying from COVID-19 as opposed to TB, ultimately resulting in a decrease in reported TB mortality. Moreover, COVID-19-related prevention measures had effectively reduced the transmission of drug-resistant TB and human immunodeficiency virus (HIV)-TB in 2020 and 2021. Compared to 2019, the World Health Organization (WHO) reported a 10.4% (n = 1903) decrease in the notified number of multidrug-resistant/rifampicin-resistant TB (MDR/RR-TB) patients in China in 2020 and a 7.8% (n = 1420) decrease in 2021, as well as decreases in the estimated number of HIV-TB patients by 7.7% (n = 1000) in 2020 and 23.1% (n = 3000) in 2021 [22]. Since drug-resistant TB and HIV-TB were the most frequent causes of death among all TB patients and spread more rapidly through the population [23,24], these reductions in HIV-TB and MDR/RR-TB patients could have a positive impact on reported TB mortality in 2020-2021.

However, we observed that, despite the initial decrease, reported mortality increased sharply after January 2022, which resulted in its long-term upward trend directly during the pandemic. The high reported mortality after January 2022 likely resulted from the disruption of TB treatment and care efforts caused by the COVID-19 pandemic. A report from England estimated that for every 30 COVID-19 deaths, there was at least one patient being admitted to hospital for non-COVID-19 reasons, but dying of the disruption of healthcare quality caused by the pandemic [25]. The WHO reported that COVID-19’s negative impact on TB care services globally had led to an estimated reduction of 1.4 million people receiving care for TB in 2020 compared to 2019 [7]. In the long-term, many TB patients could not receive standardised treatment and follow the optimal timing of treatment, leading to a sharp increase in reported mortality in the coming years after the COVID-19 outbreak. Furthermore, the lowered healthcare-seeking behaviour of TB patients might have also contributed to the delayed treatment and increased risk of mortality during the COVID-19 pandemic.

### Strengths and limitations

One of the strengths of this study was its ITS quasi-experimental design which could provide a robust analysis of intervention effect at a population level. Even without a control group, it had a strong internal validity because it could control confounding effects caused by methodological issues, such as seasonality and autocorrelation in time-series data, which could not be addressed by simply comparing the means before and after intervention [26]. As a population-level analysis, ITS also had a good external validity and its results could be extrapolated to other populations [27]. We also used 97 months' worth of data from 2015 to 2023, including 37 time points in the COVID-19 period. More time points can provide greater power to detect small changes in reported TB incidence and mortality [28].

However, our study has some limitations. First, the TB data used in the study only included confirmed and reported cases and deaths by hospitals or centres of disease prevention and control, without the latent TB infection (LTBI) patients, so we did not analyse changes in LTBI. Second, there were inevitable under-reporting or under-diagnosis proportions in the routine reporting system, and there was also a decrease in healthcare seeking-behaviour during the COVID-19 pandemic, so the reported TB incidence and mortality might be of a lower level than in reality. Finally, the design was inevitably limited in causal inference due to the lack of a control group [29]. We did not have sufficient evidence to indicate whether the same trends in TB incidence and mortality appeared in other countries or regions where COVID-19 did not occur or its impact was small.

## CONCLUSIONS

Due to the severe impact of COVID-19 on the healthcare system and healthcare-seeking behaviour among people in China, we observed a notable decrease in both reported TB incidence and mortality when COVID-19 first emerged. There further appeared to be a positive impact on reported incidence over the long-term, owing to its persistent and larger downward trend; in contrast, reported mortality increased sharply after two years of low prevalence. Further monitoring and reporting of TB incidence and mortality after the COVID-19 pandemic in China is necessary to determine the full impact of COVID-19 on TB.

## Additional material


Online Supplementary Document
