# Supplementary material for: The impact of COVID-19 pandemic on reported tuberculosis incidence and mortality in China: An interrupted time series analysis
Source: J Glob Health. 2023 Oct 13;13:06043. doi: 10.7189/jogh.13.06043 (PMC10569365; doi:10.7189/jogh.13.06043)
Supplement: Online Supplementary Document [file jogh-13-06043-s001.pdf]

The impact of COVID-19 pandemic on reported tuberculosis incidence and mortality in China: an interrupted time series analysis

Online Supplementary Document

Table S1 Complete dataset for monthly TB case notifications (incidence) and reported deaths (mortality) in China from Jan 2015 to Jan 2023

Table S2 Dataset for monthly TB predicted and reported data in China from Jan 2020 to Jan 2023

Table S3 Statistical description for TB case notifications and reported deaths (mean and variance)

Figure S1 Reported TB incidence per 100,000 person-months in the pre-COVID-19 period, Jan 1 2015 - Dec 31 2019. The red line indicated the seasonal fluctuation. TB – tuberculosis, pre-COVID-19 – before the COVID-19 pandemic.

Figure S2 Reported TB mortality per 100,000 person-months in the pre-COVID-19 period, Jan 1 2015 - Dec 31 2019. The red line indicated the seasonal fluctuation. TB – tuberculosis, pre-COVID-19 – before the COVID-19 pandemic.

Figure S3 Autocorrelation of residuals at lags for TB incidence (just adjusted to overdispersion).

Figure S4 Autocorrelation of residuals at lags for TB mortality (just adjusted to overdispersion).

Figure S5 Autocorrelation of residuals at lags for TB incidence (adjusted to overdispersion and seasonality).

Figure S6 Autocorrelation of residuals at lags for TB mortality (adjusted to overdispersion and seasonality).

Code 1 Linear regression model codes for the predicted TB case notifications and deaths.

Code 2 Model codes for the interrupted time series analysis.

Table S1. Complete dataset for monthly TB case notifications (incidence) and reported deaths (mortality) in China from Jan 2015 to Jan 2023*

Year	Month	Cases (Y1)	Deaths (Y2)	T (time)	X (level)	XT (slope)	Pop	Log pop	Ratec†	Rated†
2015	1	95151	156	1	0	0	1379860000	21.0452	0.0113	6.8957
2015	2	75541	99	2	0	0	1379860000	21.0452	0.0072	5.4745
2015	3	113242	168	3	0	0	1379860000	21.0452	0.0122	8.2068
2015	4	107997	168	4	0	0	1379860000	21.0452	0.0122	7.8267
2015	5	102067	119	5	0	0	1379860000	21.0452	0.0086	7.3969
2015	6	102388	127	6	0	0	1379860000	21.0452	0.0092	7.4202
2015	7	102986	152	7	0	0	1379860000	21.0452	0.0110	7.4635
2015	8	95746	127	8	0	0	1379860000	21.0452	0.0092	6.9388
2015	9	95081	148	9	0	0	1379860000	21.0452	0.0107	6.8906
2015	10	86024	147	10	0	0	1379860000	21.0452	0.0107	6.2343
2015	11	88115	134	11	0	0	1379860000	21.0452	0.0097	6.3858
2015	12	89818	173	12	0	0	1379860000	21.0452	0.0125	6.5092
2016	1	86181	133	13	0	0	1387790000	21.0510	0.0096	6.2099
2016	2	83527	122	14	0	0	1387790000	21.0510	0.0088	6.0187
2016	3	114627	167	15	0	0	1387790000	21.0510	0.0120	8.2597
2016	4	103397	148	16	0	0	1387790000	21.0510	0.0107	7.4505
2016	5	100500	150	17	0	0	1387790000	21.0510	0.0108	7.2417
2016	6	94064	176	18	0	0	1387790000	21.0510	0.0127	6.7780
2016	7	92793	163	19	0	0	1387790000	21.0510	0.0117	6.6864
2016	8	95924	128	20	0	0	1387790000	21.0510	0.0092	6.9120
2016	9	89342	176	21	0	0	1387790000	21.0510	0.0127	6.4377
2016	10	83092	164	22	0	0	1387790000	21.0510	0.0118	5.9874
2016	11	87963	171	23	0	0	1387790000	21.0510	0.0123	6.3384
2016	12	89609	160	24	0	0	1387790000	21.0510	0.0115	6.4570
2017	1	80911	149	25	0	0	1396215000	21.0570	0.0107	5.7950
2017	2	92037	142	26	0	0	1396215000	21.0570	0.0102	6.5919
2017	3	105633	212	27	0	0	1396215000	21.0570	0.0152	7.5657
2017	4	97296	177	28	0	0	1396215000	21.0570	0.0127	6.9686
2017	5	101628	191	29	0	0	1396215000	21.0570	0.0137	7.2788
2017	6	99001	175	30	0	0	1396215000	21.0570	0.0125	7.0907
2017	7	96471	185	31	0	0	1396215000	21.0570	0.0133	6.9095
2017	8	100076	218	32	0	0	1396215000	21.0570	0.0156	7.1677
2017	9	92494	171	33	0	0	1396215000	21.0570	0.0122	6.6246
2017	10	81554	169	34	0	0	1396215000	21.0570	0.0121	5.8411
2017	11	89976	188	35	0	0	1396215000	21.0570	0.0135	6.4443
2017	12	87630	204	36	0	0	1396215000	21.0570	0.0146	6.2763
2018	1	96125	204	37	0	0	1402760000	21.0617	0.0145	6.8526
2018	2	77224	135	38	0	0	1402760000	21.0617	0.0096	5.5051
2018	3	110124	178	39	0	0	1402760000	21.0617	0.0127	7.8505
2018	4	100054	162	40	0	0	1402760000	21.0617	0.0115	7.1327
2018	5	102063	182	41	0	0	1402760000	21.0617	0.0130	7.2759
2018	6	91603	163	42	0	0	1402760000	21.0617	0.0116	6.5302
2018	7	95338	197	43	0	0	1402760000	21.0617	0.0140	6.7965
2018	8	94232	185	44	0	0	1402760000	21.0617	0.0132	6.7176
2018	9	88302	195	45	0	0	1402760000	21.0617	0.0139	6.2949

2018	10	84680	224	46	0	0	1402760000	21.0617	0.0160	6.0367
2018	11	87709	197	47	0	0	1402760000	21.0617	0.0140	6.2526
2018	12	83205	214	48	0	0	1402760000	21.0617	0.0153	5.9315
2019	1	88597	208	49	0	0	1407745000	21.0653	0.0148	6.2935
2019	2	73096	194	50	0	0	1407745000	21.0653	0.0138	5.1924
2019	3	97866	194	51	0	0	1407745000	21.0653	0.0138	6.9520
2019	4	101191	215	52	0	0	1407745000	21.0653	0.0153	7.1882
2019	5	96106	171	53	0	0	1407745000	21.0653	0.0121	6.8269
2019	6	99555	154	54	0	0	1407745000	21.0653	0.0109	7.0719
2019	7	93318	190	55	0	0	1407745000	21.0653	0.0135	6.6289
2019	8	84304	179	56	0	0	1407745000	21.0653	0.0127	5.9886
2019	9	80973	170	57	0	0	1407745000	21.0653	0.0121	5.7520
2019	10	75123	156	58	0	0	1407745000	21.0653	0.0111	5.3364
2019	11	73000	177	59	0	0	1407745000	21.0653	0.0126	5.1856
2019	12	71631	230	60	0	0	1407745000	21.0653	0.0163	5.0884
2020	1	67682	142	61	1	0	1411100000	21.0676	0.0101	4.7964
2020	2	44933	96	62	1	1	1411100000	21.0676	0.0068	3.1843
2020	3	73427	97	63	1	2	1411100000	21.0676	0.0069	5.2035
2020	4	85684	96	64	1	3	1411100000	21.0676	0.0068	6.0721
2020	5	83385	123	65	1	4	1411100000	21.0676	0.0087	5.9092
2020	6	84952	134	66	1	5	1411100000	21.0676	0.0095	6.0203
2020	7	83101	146	67	1	6	1411100000	21.0676	0.0103	5.8891
2020	8	76423	131	68	1	7	1411100000	21.0676	0.0093	5.4158
2020	9	75409	175	69	1	8	1411100000	21.0676	0.0124	5.3440
2020	10	67843	128	70	1	9	1411100000	21.0676	0.0091	4.8078
2020	11	69640	126	71	1	10	1411100000	21.0676	0.0089	4.9352
2020	12	64097	161	72	1	11	1411100000	21.0676	0.0114	4.5423
2021	1	64813	137	73	1	12	1412360000	21.0685	0.0097	4.5890
2021	2	55425	56	74	1	13	1412360000	21.0685	0.0040	3.9243
2021	3	80803	109	75	1	14	1412360000	21.0685	0.0077	5.7211
2021	4	80548	117	76	1	15	1412360000	21.0685	0.0083	5.7031
2021	5	75243	102	77	1	16	1412360000	21.0685	0.0072	5.3275
2021	6	73884	104	78	1	17	1412360000	21.0685	0.0074	5.2312
2021	7	76648	131	79	1	18	1412360000	21.0685	0.0093	5.4269
2021	8	67966	120	80	1	19	1412360000	21.0685	0.0085	4.8122
2021	9	67812	124	81	1	20	1412360000	21.0685	0.0088	4.8013
2021	10	61391	126	82	1	21	1412360000	21.0685	0.0089	4.3467
2021	11	61753	128	83	1	22	1412360000	21.0685	0.0091	4.3723
2021	12	61788	168	84	1	23	1412360000	21.0685	0.0119	4.3748
2022	1	61697	304	85	1	25	1412175000	21.0684	0.0215	4.3689
2022	2	52596	313	86	1	26	1412175000	21.0684	0.0222	3.7245
2022	3	73110	312	87	1	27	1412175000	21.0684	0.0221	5.1771
2022	4	61185	341	88	1	28	1412175000	21.0684	0.0241	4.3327
2022	5	63590	316	89	1	29	1412175000	21.0684	0.0224	4.5030
2022	6	67901	345	90	1	30	1412175000	21.0684	0.0244	4.8083
2022	7	71422	367	91	1	31	1412175000	21.0684	0.0260	5.0576
2022	8	69019	365	92	1	32	1412175000	21.0684	0.0258	4.8874
2022	9	58638	347	93	1	33	1412175000	21.0684	0.0246	4.1523
2022	10	51125	304	94	1	34	1412175000	21.0684	0.0215	3.6203

2022	11	48352	333	95	1	35	1412175000	21.0684	0.0236	3.4239
2022	12	33951	316	96	1	36	1412175000	21.0684	0.0224	2.4042
2023	1	53730	327	97	1	37	1411750000	21.0681	0.0232	3.8059

TB – tuberculosis, Pop – population, Ratec – incidence, Rated – mortality.
*Values were number in ones.
†The units of both Ratec (incidence) and Rated (mortality) were per 100,000 person-months.

Table S2. Dataset for monthly TB predicted and reported data in China from Jan 2020 to Jan 2023*

Year	Month	Incidence				Mortality			
		Rep.cases	Pred.cases	Rep.rate	Pred.rate	Rep.deaths	Pred.deaths	Rep.rate	Pred.rate
2020	1	67682	83164	4.7964	5.8936	142	205	0.0101	0.0146
2020	2	44933	74056	3.1843	5.2481	96	174	0.0068	0.0123
2020	3	73427	102070	5.2035	7.2333	97	219	0.0069	0.0155
2020	4	85684	95758	6.0721	6.7861	96	209	0.0068	0.0148
2020	5	83385	94244	5.9092	6.6788	123	198	0.0087	0.0140
2020	6	84952	91093	6.0203	6.4555	134	194	0.0095	0.0138
2020	7	83101	89952	5.8891	6.3746	146	213	0.0103	0.0151
2020	8	76423	87828	5.4158	6.2241	131	203	0.0093	0.0144
2020	9	75409	83010	5.3440	5.8826	175	207	0.0124	0.0147
2020	10	67843	75866	4.8078	5.3764	128	207	0.0091	0.0147
2020	11	69640	79124	4.9352	5.6072	126	209	0.0089	0.0148
2020	12	64097	78150	4.5423	5.5382	161	232	0.0114	0.0164
2021	1	64813	81088	4.5890	5.7413	137	217	0.0097	0.0154
2021	2	55425	71980	3.9243	5.0964	56	186	0.0040	0.0131
2021	3	80803	99993	5.7211	7.0799	109	231	0.0077	0.0164
2021	4	80548	93682	5.7031	6.6330	117	221	0.0083	0.0157
2021	5	75243	92168	5.3275	6.5258	102	210	0.0072	0.0149
2021	6	73884	89017	5.2312	6.3027	104	206	0.0074	0.0146
2021	7	76648	87876	5.4269	6.2219	131	225	0.0093	0.0159
2021	8	67966	85751	4.8122	6.0715	120	215	0.0085	0.0152
2021	9	67812	80933	4.8013	5.7304	124	219	0.0088	0.0155
2021	10	61391	73790	4.3467	5.2246	126	219	0.0089	0.0155
2021	11	61753	77048	4.3723	5.4552	128	221	0.0091	0.0156
2021	12	61788	76074	4.3748	5.3863	168	243	0.0119	0.0172
2022	1	61697	79012	4.3689	5.5950	304	229	0.0215	0.0162
2022	2	52596	69904	3.7245	4.9501	313	197	0.0222	0.0140
2022	3	73110	97917	5.1771	6.9338	312	243	0.0221	0.0172
2022	4	61185	91606	4.3327	6.4868	341	233	0.0241	0.0165
2022	5	63590	90091	4.5030	6.3796	316	222	0.0224	0.0157
2022	6	67901	86941	4.8083	6.1565	345	218	0.0244	0.0154
2022	7	71422	85800	5.0576	6.0757	367	236	0.0260	0.0167
2022	8	69019	83675	4.8874	5.9253	365	226	0.0258	0.0160
2022	9	58638	78857	4.1523	5.5841	347	231	0.0246	0.0164
2022	10	51125	71713	3.6203	5.0782	304	231	0.0215	0.0164
2022	11	48352	74971	3.4239	5.3089	333	232	0.0236	0.0165
2022	12	33951	73997	2.4042	5.2400	316	255	0.0224	0.0181
2023	1	53730	76935	3.8059	5.4496	327	241	0.0232	0.0171

TB – tuberculosis, Rep – reported, Pred – predicted.

*Cases and deaths were number in ones, and rates were number in per 100,000 person-months.

Table S3. Statistical description for TB case notifications and reported deaths (mean and variance)

Variable	Stage	Mean*	Variance*
case notifications	X = 0(pre-COVID-19)	92421.68	97381094.93
	X = 1(during-COVID-19)	66782.86	138652744.51
deaths	X = 0(pre-COVID-19)	170.52	802.36
	X = 1(during-COVID-19)	196.41	10514.45

COVID-19 – coronavirus disease-2019, TB – tuberculosis, pre-COVID-19 – before the COVID-19 pandemic, during-COVID-19 – during the COVID-19 pandemic.
*Values were number in ones.

Figure S1. Reported TB incidence per 100,000 person-months in the pre-COVID-19 period, Jan 1 2015 - Dec 31 2019. The red line indicated the seasonal fluctuation. TB – tuberculosis, pre-COVID-19 – before the COVID-19 pandemic.

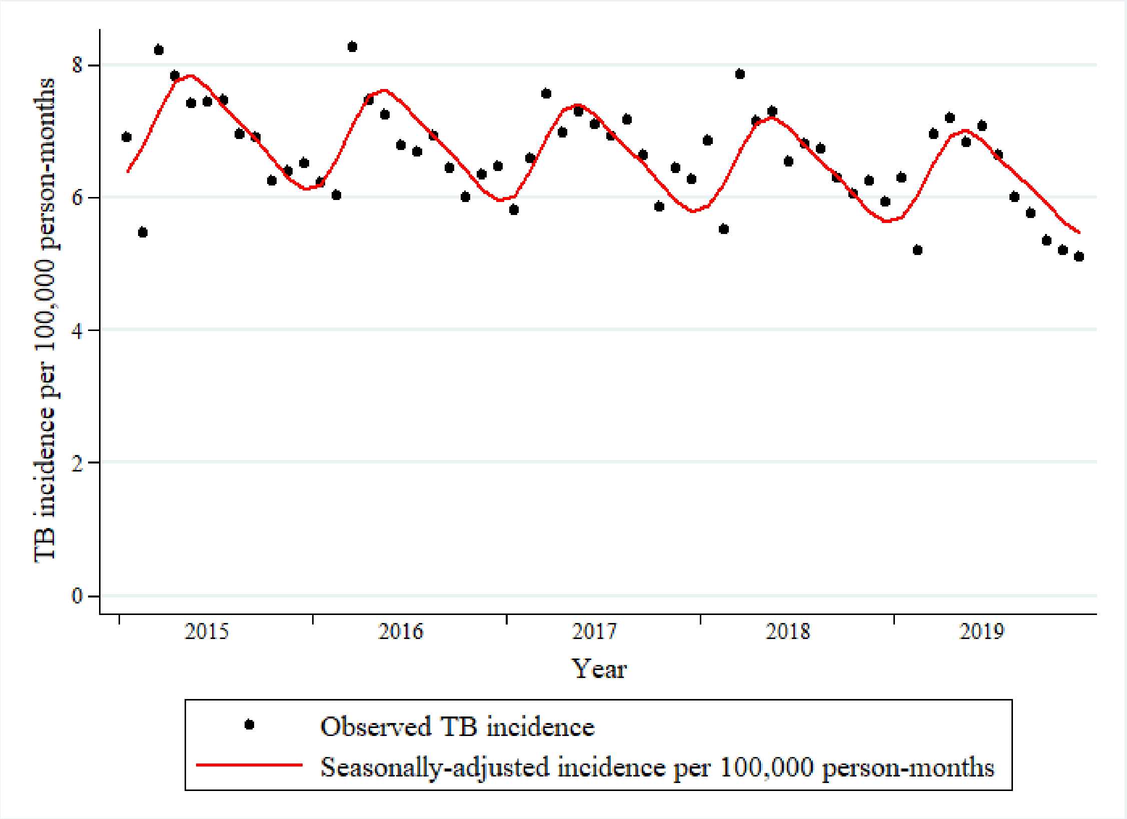

Figure S2. Reported TB mortality per 100,000 person-months in the pre-COVID-19 period, Jan 1 2015 - Dec 31 2019. The red line indicated the seasonal fluctuation. TB – tuberculosis, pre-COVID-19 – before the COVID-19 pandemic.

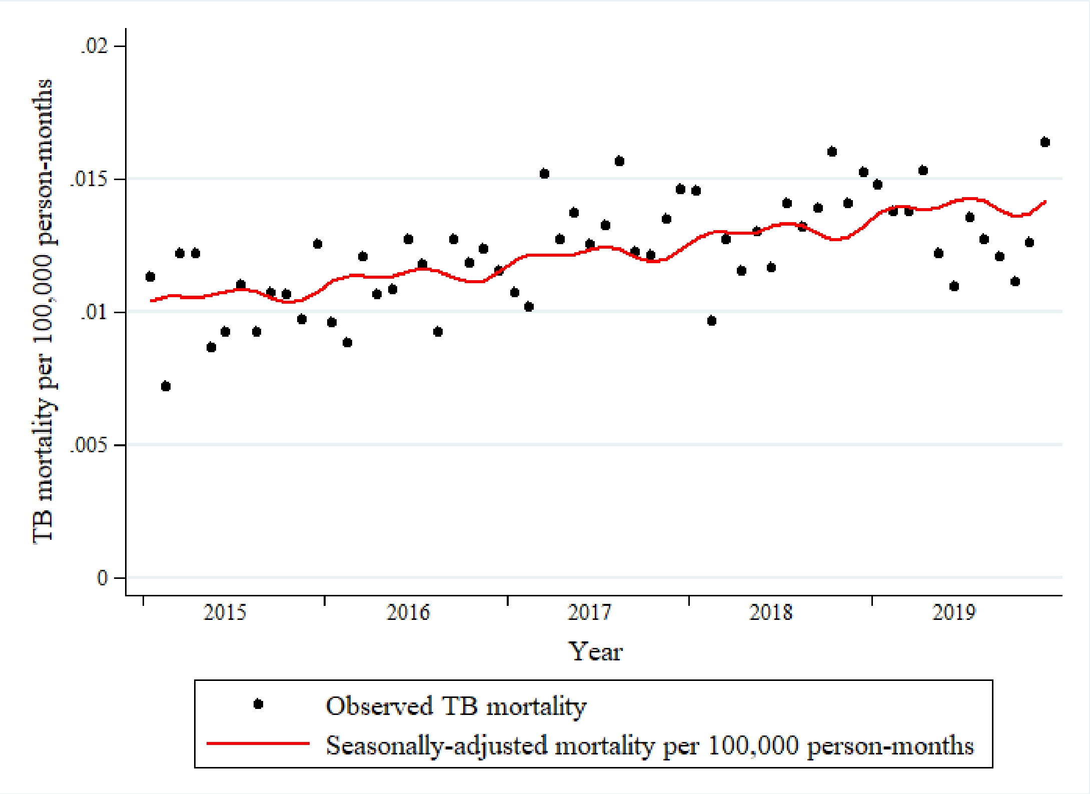

Figure S3. Autocorrelation of residuals at lags for TB incidence (just adjusted to overdispersion).

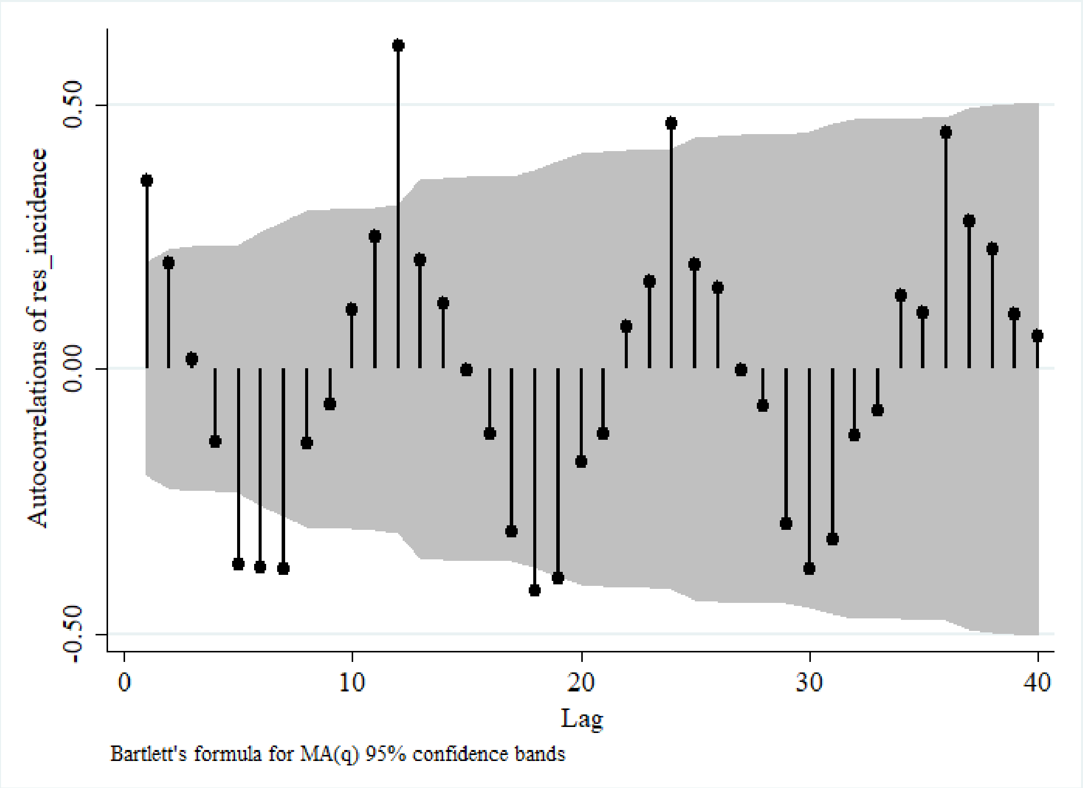

Figure S4. Autocorrelation of residuals at lags for TB mortality (just adjusted to overdispersion).

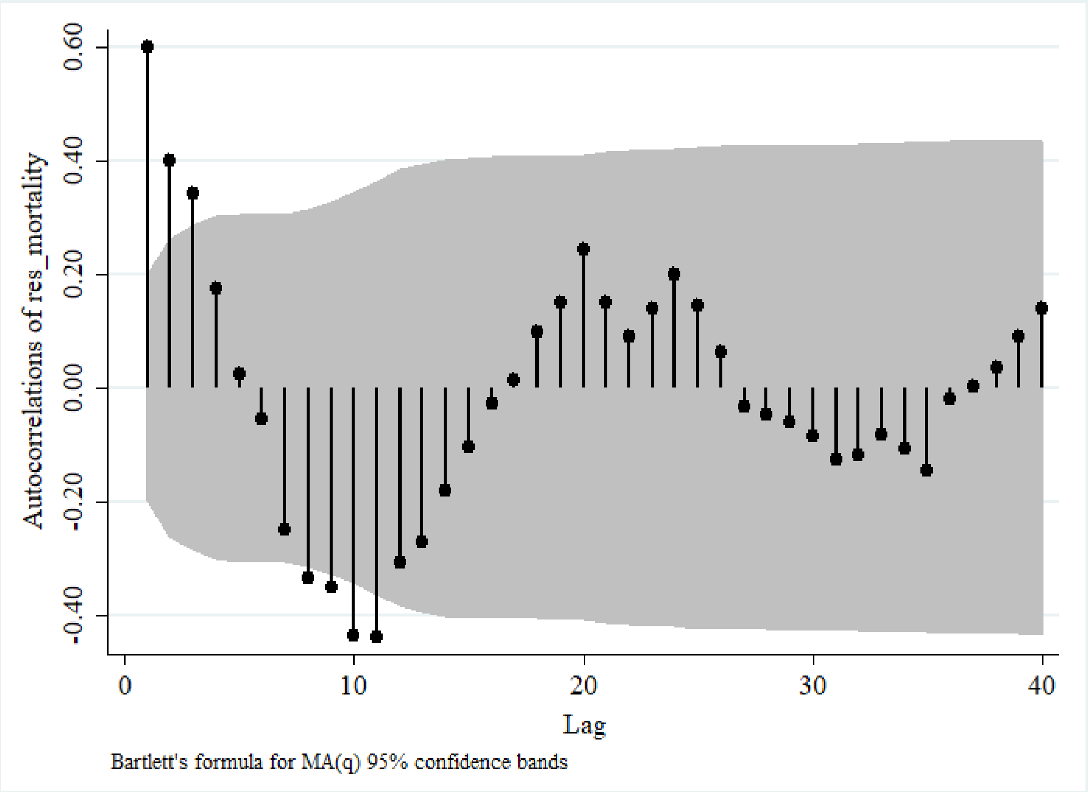

Figure S5. Autocorrelation of residuals at lags for TB incidence (adjusted to overdispersion and seasonality).

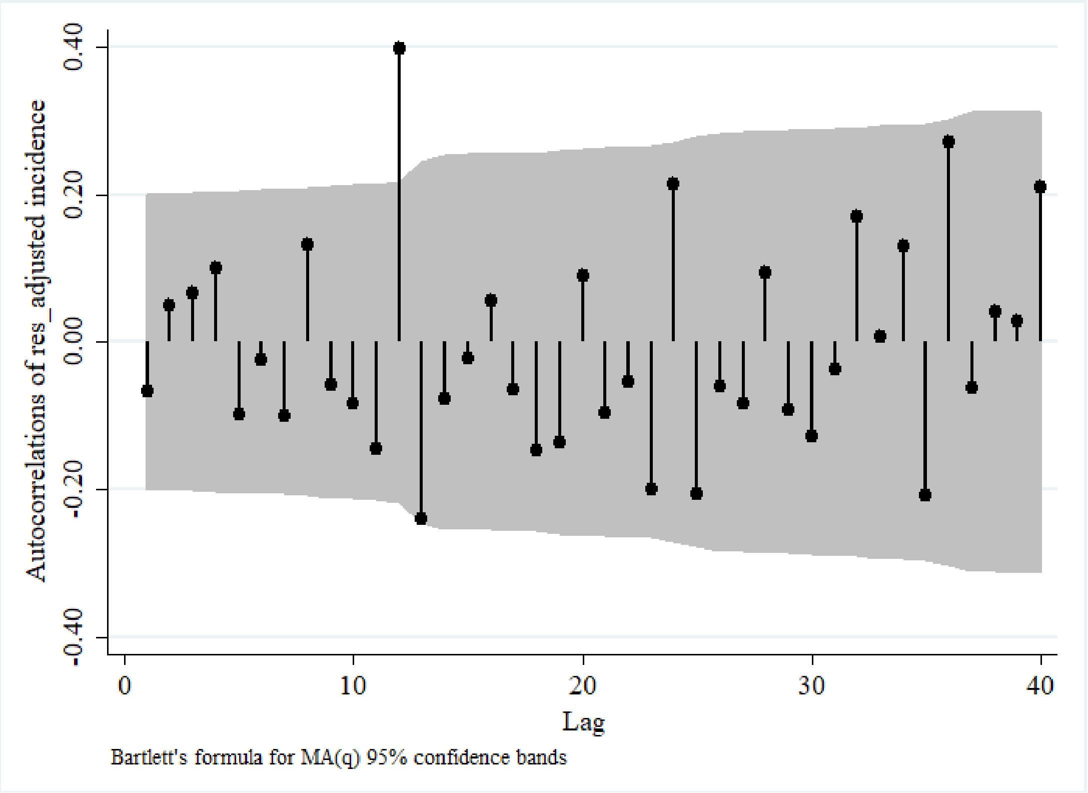

Figure S6. Autocorrelation of residuals at lags for TB mortality (adjusted to overdispersion and seasonality).

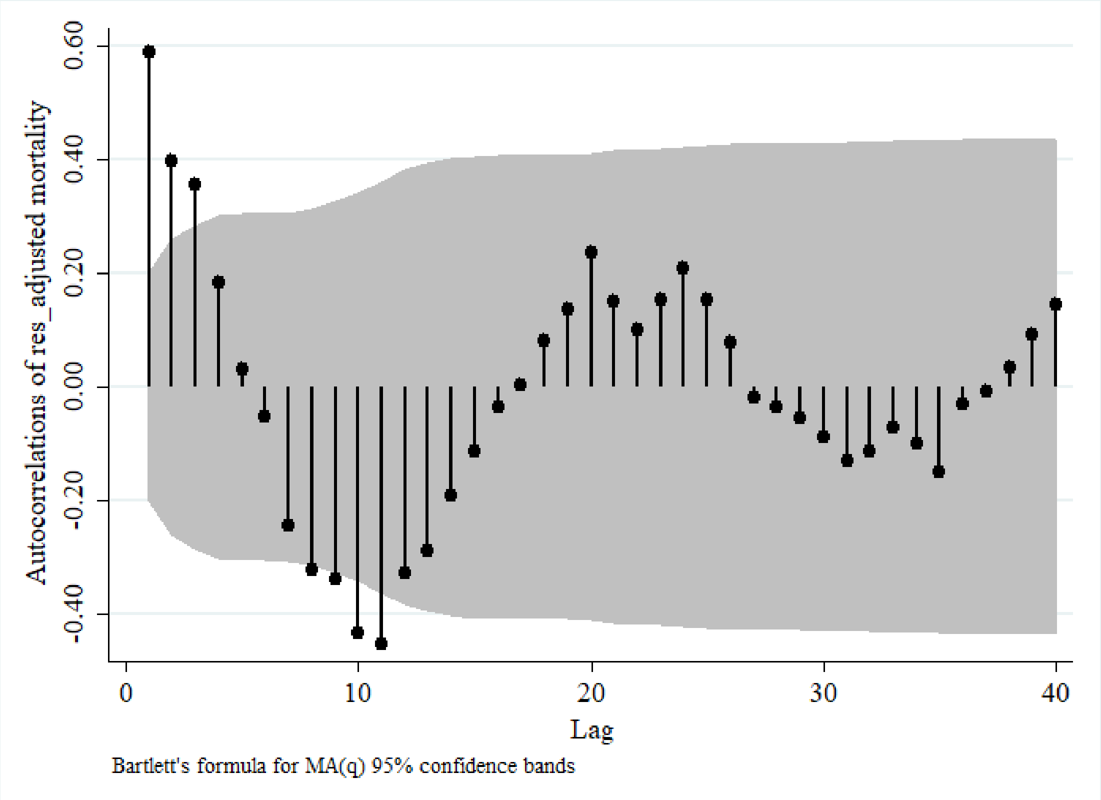

Code 1. Linear regression model codes for the predicted TB case notifications and deaths.

```
import numpy as np
import pandas as pd
from sklearn.linear_model import LinearRegression
data = pd.read_excel('table S2.xlsx')
data.to_csv(' table S2.csv',index=False)
table S2_data = pd.read_csv(' table S2.csv')
X = np.array(table S2_data)
# In this data, columns 0-3 were year, month, cases and deaths, respectively.

# Fit regression model on 2015-2019 data.
def predict(X, verbose=False, excess_begin=None):
    ind = (X[:,0] < 2020)
    m = np.max(X[ind,1])
    onehot = np.zeros((np.sum(ind), m))
    for i,k in enumerate(X[ind,1]):
```

```
            onehot[i,k-1] = 1
        predictors = np.concatenate((X[ind,:1], onehot), axis=1)
        reg = LinearRegression(fit_intercept=False).fit(predictors, X[ind,3])
        print('coef--->',reg.coef_,'\n','inte-->',reg.intercept_)
        print('test--->',predictors.shape,'\n',predictors,
                X[ind,3])
        if verbose:
            est = sm.OLS(X[ind,3], predictors).fit()
            print(est.summary())
        # If calculating the predicted deaths, simply replace all of the X[ind,3] above with X[ind,4].

        # Compute 2020 predicted data.
        ind2 = X[:,0] == 2020
        predictors2020 = np.concatenate((np.ones((m,1))*2020, np.eye(m)), axis=1)
        pred.cases = reg.predict(predictors2020)
return pred.cases

        # Compute 2021 predicted data.
        ind3 = X[:,0] == 2021
        predictors2021 = np.concatenate((np.ones((m,1))*2021, np.eye(m)), axis=1)
        pred.cases 2021 = reg.predict(predictors2021)
return pred.cases 2021

        # Compute 2022 predicted data.
        ind4 = X[:,0] == 2022
        predictors2022 = np.concatenate((np.ones((m,1))*2022, np.eye(m)), axis=1)
        pred.cases 2022 = reg.predict(predictors2022)
return pred.cases 2022

        # Compute 2023 predicted data.
        ind4 = X[:,0] == 2023
        predictors2023 = np.concatenate((np.ones((m,1))*2023, np.eye(m)), axis=1)
        pred.cases 2023 = reg.predict(predictors2023)
return pred.cases 2023
# Similarly, if calculating the predicted deaths, replace all of the pred.cases above with pred.deaths.
```

```
# print(X,type(X))
predict(X)
```

Code 2. Model codes for the interrupted time series analysis.

```
###Statistical description before and during the intervention:
summ,detail
bysort X:summ deaths
bysort X:summ rated
bysort X:summ cases
bysort X:summ ratec

###Log transform the population:
gen logpop = log(pop)

###Compute the predicted incidence and mortality (/100,000 person-months):
gen pred1 = pred.deaths/pop*100000
gen predc1 = pred.cases/pop*100000

###We proposed Poisson regression models with the levels (X) and slopes (XT) changed, due to the rate do not follow a Poisson distribution, we directly used the count data as the dependent variable and the log-transformed population as the offset variable to transform the outcome to rate:
glm deaths X XT T , family(poisson) link(log) offset(logpop) eform
glm cases X XT T , family(poisson) link(log) offset(logpop) eform

###Scale parameter set to x2 to adjust to overdispersion in the model:
glm deaths X XT T , family(poisson) link(log) offset(logpop) scale(x2) eform
glm cases X XT T , family(poisson) link(log) offset(logpop) scale(x2) eform

###Checked residual plot and autocorrelation plot of residuals:
predict res,r
tsset T
ac res

###Built Fourier terms function:
```

gen degrees=(T/12)*360

fourier degrees, n(2)

###Added Fourier terms to Poisson model to adjust to seasonality:

glm deaths X XT cos* sin* T , family(poisson) link(log) offset(logpop) scale(x2) eform

glm cases X XT cos* sin* T , family(poisson) link(log) offset(logpop) scale(x2) eform

###Checked autocorrelation again:

predict res2,r

tsset T

ac res2

###Generate the predictor variables of seasonally-adjusted models:

predict pred2,nooffset

predict predc2,nooffset

###Transform the predictor variables into rates /100,000 person-months:

foreach var of varlist pred2 predc2 {

replace `var' = `var'*100000

}

###Scatterplot of pre-exposure TB incidence:

twoway(scatter ratec T)(line predc2 T, color(red))if X==0,title("TB incidence /100000 person-months, Jan 1 2015 - Dec 31 2019")ytitle(Incidence /100000 person-months) yscale(range(0.)) ylabel(#5,labsize(small)angle(horizontal))xtick(0.5(12)48.5)xlabel(6"2015"18"2016"30"2017"42"2018"54"2019",noticks labsize(small))xtitle(Year)

###Scatterplot of pre-exposure TB mortality:

twoway(scatter rated T)(line pred2 T, color(red))if X==0,title("TB mortality /100000 person-months, Jan 1 2015 - Dec 31 2019")ytitle(Mortality /100000 person-months) yscale(range(0.)) ylabel(#5,labsize(small)angle(horizontal))xtick(0.5(12)48.5)xlabel(6"2015"18"2016"30"2017"42"2018"54"2019",noticks labsize(small))xtitle(Year)

###Added counterfactual to plot:

twoway(scatter rated T)(line pred2 T, color(red))(line pred1 T, lcolor(red) lpattern(dash)),title("TB mortality, Jan 1 2015 - Jan 1 2023") ytitle(Mortality) yscale(range(0.))
ylabel(#5,labsize(small)angle(horizontal))xtick(0.5(12)96.5)xlabel(6"2015"18"2016"30"2017"42"2018"54"2

```
019"66"2020"78"2021"90"2022"102"2023",noticks labsize(small))xtitle(Year)xline(60.5)
```

```
twoway(scatter ratec T)(line predc2 T, color(red))(line predc1 T, lcolor(red) lpattern(dash)),title("TB incidence, Jan 1 2015- Jan 1 2023") ytitle(Incidence) yscale(range(0.)) ylabel(#5,labsize(small)angle(horizontal))xtick(0.5(12)96.5)xlabel(6"2015"18"2016"30"2017"42"2018"54"2019"66"2020"78"2021"90"2022"102"2023",noticks labsize(small))xtitle(Year)xline(60.5)
```

###Sensitivity analyses, adjusted to seasonality:

```
glm cases X XT cos* sin* T, family(nbinomial 1) link(log) offset(logpop) scale(x2) eform
glm deaths X XT cos* sin* T, family(nbinomial 1) link(log) offset(logpop) scale(x2) eform
```

###Sensitivity analyses, adjusted to seasonality and autocorrelation:

```
glm deaths X XT cos* sin* T, family(nbinomial 1) link(log) offset(logpop) eform vce(hac nwest 4)
glm case X XT cos* sin* T, family(nbinomial 1) link(log) offset(logpop) eform vce(hac nwest 4)
```

###Plotted a straight line as if all months were the average to produce a 'deseasonalised' trend, in order to clearly see the change graphically in the seasonally adjusted model:

```
egen avg_cos_1=mean(cos_1)
egen avg_sin_1=mean(sin_1)
egen avg_cos_2=mean(cos_2)
egen avg_sin_2=mean(sin_2)
drop cos* sin*
rename avg_cos_1 cos_1
rename avg_sin_1 sin_1
rename avg_sin_2 sin_2
rename avg_cos_2 cos_2
```

###Predicted and added a line of 'deseasonalised' trend to the plot:

```
predict pred3, nooffset
predict predc3, nooffset

foreach var of varlist predc3 pred3{
replace `var' = `var'*100000
}

twoway(scatter rated T)(line pred2 T, color(red))(line pred3 T, color(red) lpattern(dash)), ytitle("TB mortality
```

/100000 person-months") yscale(range(0.)) ylabel(#5,labsize(small)angle(horizontal))xtick(0.5(12)72.5)xlabel(6"2015"18"2016"30"2017"42"2018"54"2019"66"2020"78"2021"90"2022"102"2023", noticks labsize(small)) xtitle(Year) xline(60.5) legend(label(1 "Observed TB mortality") label(2 "Seasonally-adjusted mortality per 100000 person") label(3 "Unadjusted mortality per 100000 person") order(1 3 2) cols(1))

twoway(scatter ratec T)(line predc2 T, color(red))(line predc3 T, color(red) lpattern(dash)), ytitle("TB incidence /100000 person-months") yscale(range(0.)) ylabel(#5,labsize(small)angle(horizontal))xtick(0.5(12)72.5)xlabel(6"2015"18"2016"30"2017"42"2018"54"2019"66"2020"78"2021"90"2022"102"2023", noticks labsize(small)) xtitle(Year) xline(60.5) legend(label(1 "Observed TB incidence") label(2 "Seasonally-adjusted incidence per 100000 person") label(3 "Unadjusted incidence per 100000 person") order(1 3 2) cols(1))
